# Severity of Mitral Valve Degeneration Is Associated with Chromosome 15 Loci in Whippet Dogs

**DOI:** 10.1371/journal.pone.0141234

**Published:** 2015-10-28

**Authors:** Joshua A. Stern, Weihow Hsue, Kun-Ho Song, Eric S. Ontiveros, Virginia Luis Fuentes, Rebecca L. Stepien

**Affiliations:** 1 University of California Davis, Department of Medicine & Epidemiology, One Shields Ave, Davis, California, 95616, United States of America; 2 Clinical Science and Services, Royal Veterinary College, Hawkshead Lane, North Mymms, Hertfordshire, AL9 7TA, United Kingdom; 3 University of Wisconsin, School of Veterinary Medicine, Department of Medical Sciences (Cardiology), 2015 Linden Drive, Madison, Wisconsin, 53706, United States of America; GI Lab, UNITED STATES

## Abstract

Mitral valve degeneration (MVD) is the most common form of heart disease in dogs, frequently leading to left-sided congestive heart failure and cardiac mortality. Although breed-specific disease characteristics and overrepresentation point towards a genetic origin for MVD, a causative mutation and complete molecular pathogenesis are unknown. Whippet dogs are overrepresented in incidence of MVD, suggesting an inherited component in this breed. Expressivity of this condition is variable with some dogs showing evidence of more severe disease at earlier ages than other dogs. This phenomenon makes a traditional case versus control genetic study prone to phenotyping error. This study sought to avoid these common pitfalls by identifying genetic loci associated with severity of MVD in Whippets through a genome-wide association study (GWAS). 138 Whippet dogs were characterized for MVD by echocardiographic examination and a novel disease severity score was developed and adjusted for age in each subject. Single nucleotide polymorphism (SNP) genotype data (170k Illumina CanineHD SnpChip) was obtained for DNA isolated from blood of each study subject. Continuous variable genome wide association was performed after correction for population stratification by efficient mixed model association expedited (EMMAX) in 130 dogs. A genome wide significant association was identified on chromosome 15 (peak locus 57,770,326; P_adj_ = 0.049) and secondary loci of suggestive association were identified on chromosome 2 (peak locus 37,628,875; P_adj_ = 0.079). Positional candidate genes were identified within the primary and secondary loci including follistatin-related protein 5 precursor (FSTL5) and Rho GTPase-activating protein 26 (ARHGAP26). These results support the hypothesis that severity of MVD in whippets has a genetic basis and warrants further study by either candidate gene sequencing or next-generation techniques.

## Introduction

Mitral valve degeneration (MVD) is the most common form of heart disease in dogs, accounting for 75% of all dogs with cardiac disease [1]. The pathology of this disease in the dog resembles that of primary mitral valve prolapse (MVP) of humans in some respects [2]. MVP is a common cardiovascular abnormality, occurring in 2–8% of the human population [3]. In humans, MVP is associated with clinically-important sequela, including mitral regurgitation, bacterial endocarditis, congestive heart failure, atrial fibrillation, and sudden death [4].

In dogs, MVD is an acquired disease of advanced age [5]. The disease is characterized by chronic progressive degenerative lesions of the mitral valve, where the valve leaflets become thickened, develop poor coaptation, and may prolapse during systole into the left atrium [6]. These valve lesions result in mitral regurgitation, elevated left atrial pressure and, ultimately in some dogs, left-sided congestive heart failure and cardiac mortality [7].

MVD can occur in all breeds, but is most prevalent in small to medium-sized dog breeds such as Cavalier King Charles Spaniels, Dachshund, Miniature Poodle, Maltese, Pomeranian, Yorkshire Terrier and Chihuahua [8]. One study, recently reported 2 loci weakly associated with development of MVD by GWAS in the Cavalier King Charles Spaniel breed [9]. Some over-represented breeds have an incredibly high disease frequency with dogs such as the CKCS showing >80 affection rates in populations >8yrs of age [10]. A clear problem exists when considering MVD as a disease amenable to case vs. control genome wide association analysis (GWAS). It is well documented that canine MVD has age-related penetrance making it difficult to accurately phenotype an individual as a control. Determining an age cutoff that accurately predicts MVD development is challenging and provides an imperfect control population hence underscoring the relative failures of previous techniques [9,11].

In Whippets, previous publications and an ongoing longitudinal study of healthy and MVD affected dogs have demonstrated high frequency of MVD and a comparably early age of onset in some dogs supporting its plausible genetic origin [12–14]. The purpose of this study was to identify a genetic locus associated with the development or severity of MVD in Whippets.

## Materials and Methods

### Ethics Statement

This study was performed on 138 normal and MVD-affected, client-owned Whippet dogs. Owner consent was obtained prior to evaluation and blood collection. The University of California, Davis Animal Care and Use Committee approved the protocol.

**Fig 1:**
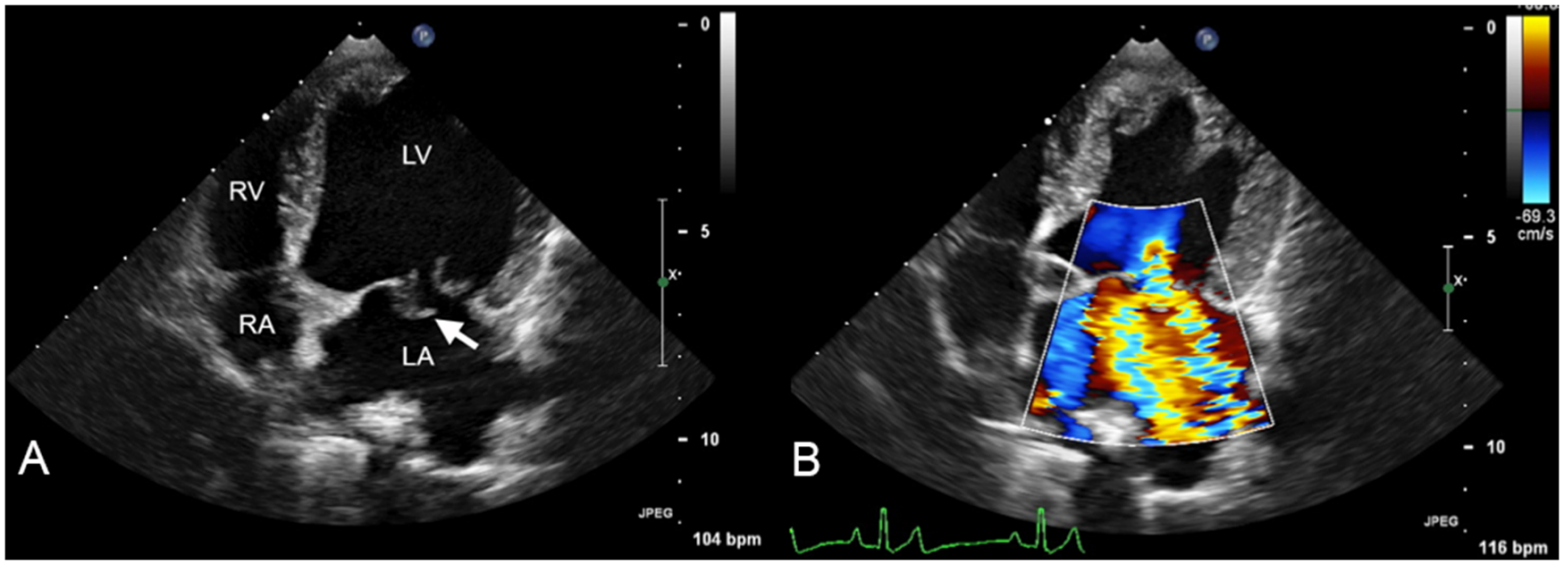
A two-dimensional (A) and two-dimensional with color Doppler (B) echocardiogram from a dog are displayed. The view is left apical four-chamber showing the left ventricle (LV), left atrium (LA), right ventricle (RV) and right atrium (RA). The mitral valve is denoted by the white arrow in image A. This image represent a dog with severe mitral valve prolapse as it is observed to buckle back into the LA during systole. Severe mitral valve regurgitation and chamber enlargement are also apparent, yielding a disease severity score of 6 in this dog.

Study population, clinical evaluation and disease scoring:

138 Whippet dogs were enrolled in this study as a genetic arm to an ongoing longitudinal clinical investigation. All dogs received cardiac auscultation, a two-dimensional (2D), M-mode and Doppler echocardiogram and had 2mL of blood collected in EDTA for future DNA extraction. Disease phenotype was detailed using results of auscultation and echocardiographic information. Heart murmurs were graded as 0-6/6. Cumulative echocardiographic MVD severity score was calculated for each dog on a scale of 0-6/6. This score was derived based upon the noted presence or severity of pre-determined echocardiographic findings as noted by the reporting cardiologist including: mitral valve prolapse, mild mitral valve regurgitation (MR), and chamber enlargement (left atrium, left ventricle or both; Table 1). An example of how the scoring system is generated by echocardiogram is demonstrated in Fig 1.

**Table 1:** Cumulative Echocardiographic Score System (Maximum of 6).

Echocardiographic Variable	Score
Mitral Valve Prolapse	Absent = 0
	Mild or Moderate = 1
	Severe = 3
Mitral Valve Regurgitation	Absent = 0
	Mild = 1
	Moderate and/or Multiple Jets = 2
	Severe = 3
Left Atrial or Ventricular Enlargement	Absent = 0
	Present = 1

Degenerative valve disease in a highly affected population is a challenging population to consider the use of standard case vs. control GWAS models. The age-related penetrance of MVD makes identification of true control dogs difficult as it is impossible to determine if a patient will develop disease if they live long enough. A continuous variable association was considered and used to take full advantage of the samples available in this study and deal with the difficulties of age-related penetrance.

A continuous variable was constructed to account for the age-related nature of disease severity, where younger dogs with MVD would be considered a more severe disease variant than older dogs with the same level of degenerative change. The age of 5 years was chosen as a pivotal point in the breed, where dogs less than 5 years demonstrating clear evidence of disease would be considered the most severely affected animals. To account for age in this construct, the MVD severity score (made up of discrete variables totaling a maximum of 6 points) was multiplied by 5 and then divided by the patient’s age in years, yielding a continuous variable. For example, a 4-year-old dog with a MVD severity score of 4 would be assigned the final continuous variable value of 5 [(4*5)/4]. This continuous variable scoring was carried out for each dog using their age at time of echocardiographic evaluation in years. This scale was derived for use in this study and the ongoing longitudinal study of MVD in Whippets. To clarify, the multiplication by 5 in this model produces a linear scaling effect that makes the numbers easier to manage and understand in a clinical setting and does not affect association results.

The routine echocardiographic technique is standardized as part of an ongoing longitudinal study of mitral valve degeneration in Whippets. The echocardiographic image acquisition protocol and measurement methodology is derived from previously described methods [15,16].

Mitral valve regurgitation severity was judged subjectively based upon a color Doppler surface area methodology as previously described [17]. The color Doppler signal was optimized by adjusting the pulsed repetition frequency to a gain level just below the point where speckle occurs [17]. Mild mitral valve regurgitation was characterized as a single regurgitant jet with a regurgitant jet that occupied <30% of the atrium. Moderate regurgitation could include multiple jets and/or a jet that occupied between 30 and 70% of the left atrium. Severe regurgitation occupied >70% of the left atrium.

Severity of mitral valve prolapse was judged according to Boon [17]. Briefly stated, mild or moderate prolapse was defined as buckling of either or both of the mitral valve leaflets into the left atrium beyond the point of the mitral valve annulus. Severe mitral valve prolapse was defined as bucking of the mitral valve during systole further into the atrium beyond the point of the dense echogenic area of the lower interatrial septum or atrioventricular junction [17]. Additionally the presence of visible chordae rupture or flail mitral valve leaflets were scored as severe prolapse.

### Blood collection and DNA extraction

DNA was extracted from EDTA blood samples using a commercially available kit (Puregene, Gentra Systems, Minneapolis, MN), according to the manufacture’s protocol.

Genotyping and quality control:

300ng of high quality (260/280 ratio of 1.8–1.9) DNA was submitted and genotyped using the 170k Illumina CanineHD BeadChip (Illumina, San Diego, CA). Raw genotype data was processed for quality control using commercially available software (Golden Helix, Bozeman, MT, USA) as follows: SNPs were excluded if they had a call rate of < 95% or if they deviated significantly from Hardy-Weinberg equilibrium (*P* < 0.001) or if they had low minor allele frequency (< 8%). SNPs residing on the X chromosome were excluded, as there is no indication for an X-linked pattern of inheritance in the pathogenesis of canine MVD. Individuals were excluded if either the quantity of isolated DNA was insufficient for the array or the genotyping rate per individual was <95%.

### Genome-wide Association, adjustment for population stratification

Continuous variable association was carried out using commercially available software (Golden Helix, Bozeman, MT, USA) with the calculated MVD severity score normalized to age as the phenotypic variable of interest. Multiple testing correction was performed in order to determine genome wide significance. The robust Bonferroni correction was utilized and genome-wide significance was set at a P value of 0.05.

A Quantile-Quantile (Q-Q) plot was constructed using observed *P*-values against expected *P*-values. A genome inflation factor (λ) was calculated by dividing median χ^2^ statistics by 0.456 to assess potential population stratification of the study subjects [18]. The effect of population stratification was assessed by examining the Q-Q plot for deviation of the *p*-value from the null hypothesis. If λ>1, efficient mixed model association expedited (EMMAX) was used to correct population stratification by applying a kinship matrix and its correction was reevaluated by a second analysis of the post-EMMAX Q-Q plot and new continuous variable association results [19].

The SNPs most significantly associated with MVD from the continuous variable analysis are detailed by location in CanFam 3.1 and test statistics. Any SNP meeting or approaching the apriori genome-wide (Bonferroni) significance was investigated for proximity to annotated genes in CanFam3.1. The described gene function and any published link to cardiac disease in any species was evaluated and reported.

In order to demonstrate the value of the continuous variable approach, a simple association using case vs. control methodology was also performed. Cases (n = 26) were described as dogs with a disease severity score of 3 or greater at any age while controls (n = 20) were dogs with no evidence of mitral valve regurgitation and an age of at least 5 years.

## Results

**Fig 2:**
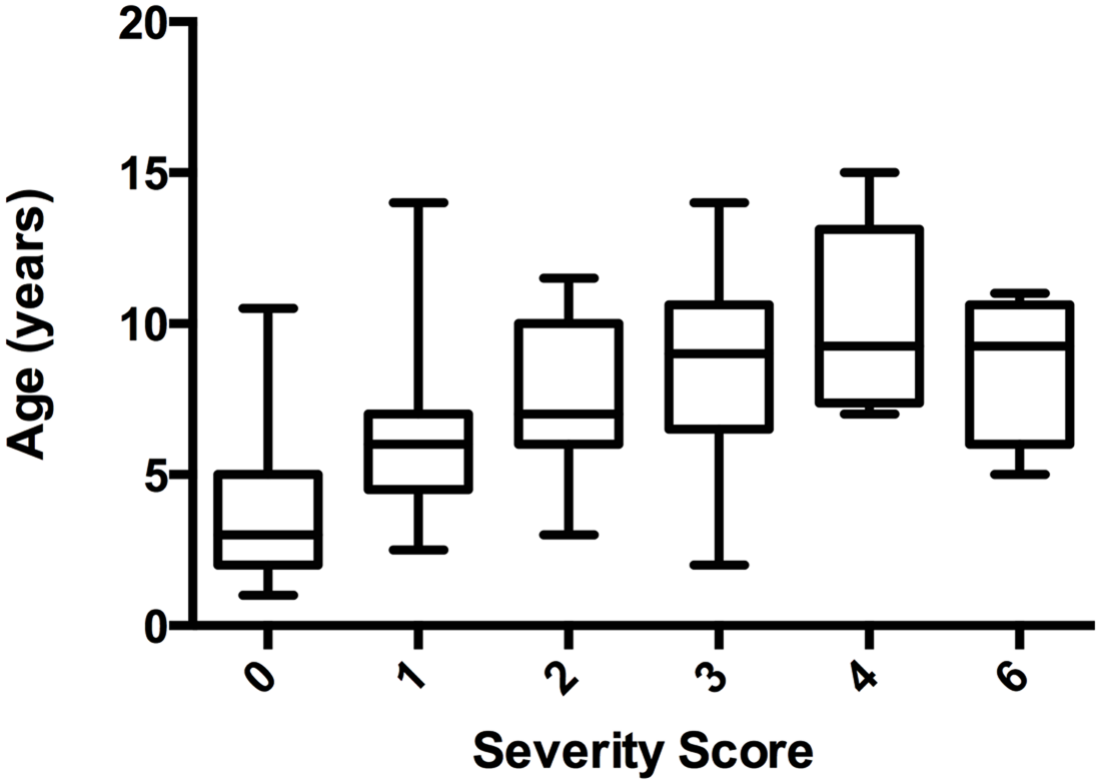
A box and whiskers plot displays the median and range of ages across each severity score group that was present within this study.

138 Whippets were prospectively enrolled with 130 remaining for genotype association after exclusion of inadequate-quality DNA samples and low genotyping rate. Sex distribution of the remaining 130 dogs was approximately equal with 57 males, 59 females, 1 castrated male and 13 spayed females. The median age was 4.5 years at time of echocardiographic evaluation with an interquartile range of 2.5 and 7.1 years respectively. The youngest dog evaluated was 1 year and the oldest 15 years of age. Of 130 dogs the echocardiogram severity score prior to age indexing was distributed as follows: 83 dogs scored 0; 13 dogs scored 1; 14 dogs scored 2; 10 dogs scored 3; 6 dogs scored 4; 0 dogs scored 5 and 4 dogs scored 6. The distribution of echocardiographic severity score and age is depicted in Fig 2.

**Fig 3:**
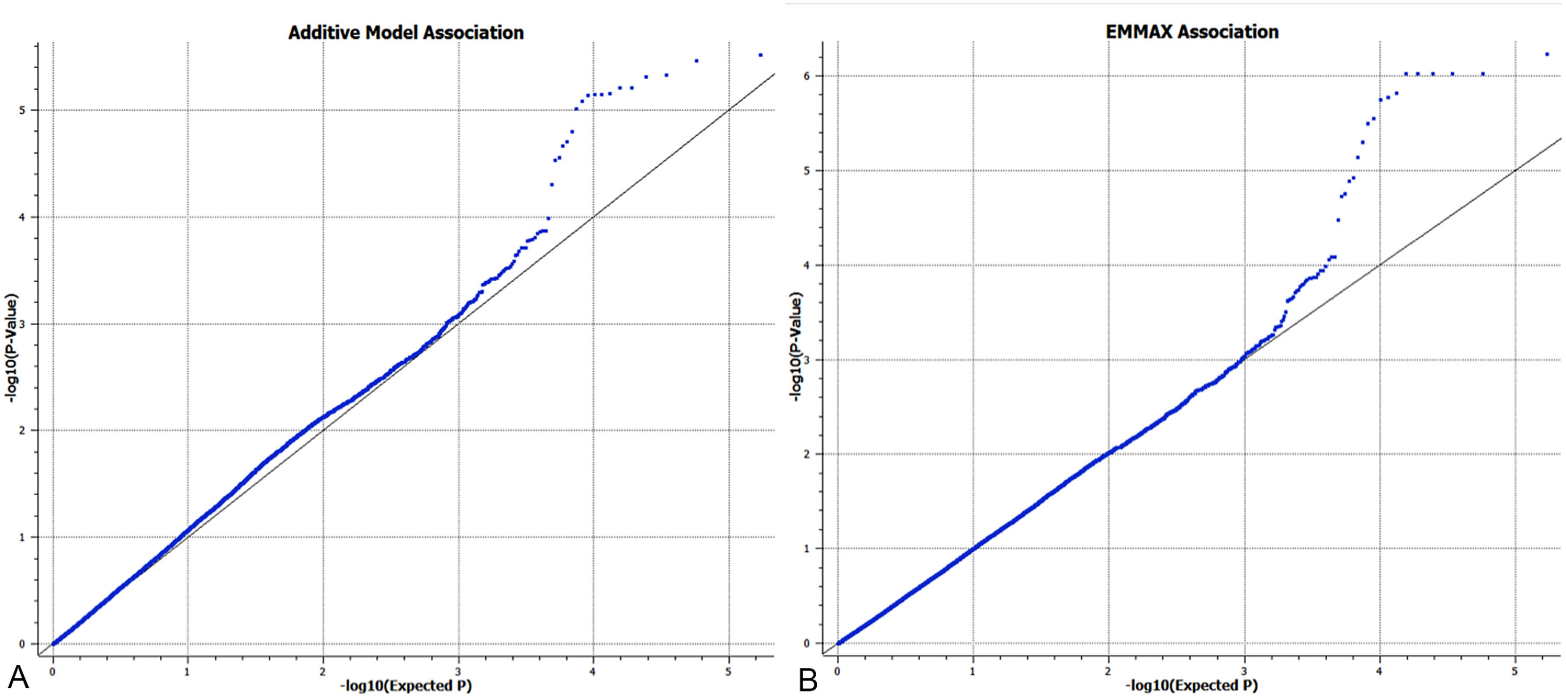
A) Q-Q plot from the continuous variable association with λ = 1.07 demonstrating mild population stratification as seen by the continual deviation from the line (consistent obtained P values deviate from those which are expected). B) Q-Q plot from the efficient mixed model association expedited (EMMAX) analysis in which the deviation from expected P values is corrected yielding a single, pronounced deviation from expect P values, consistent with the significant association results obtained.

**Fig 4:**
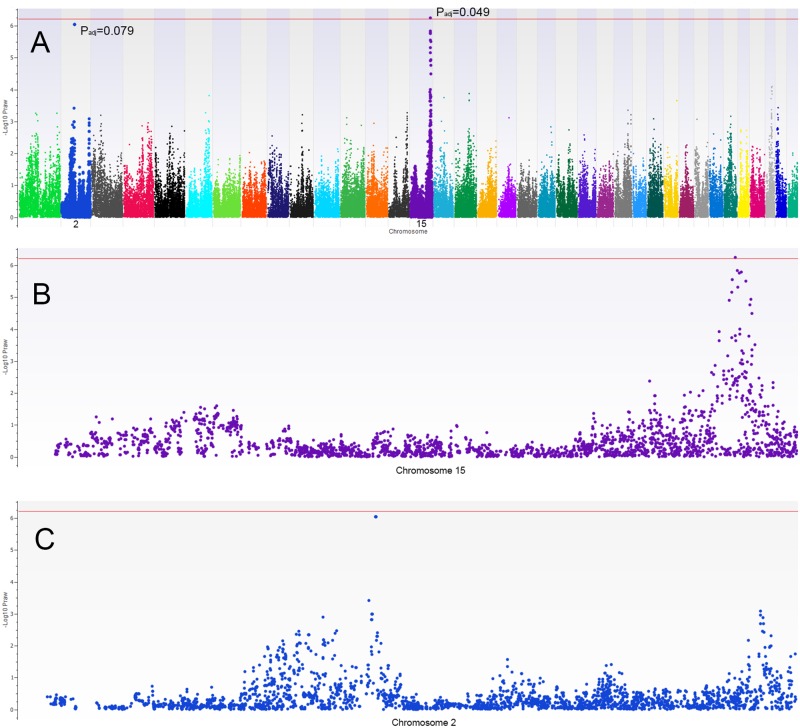
A) Manhattan plot demonstrating the genotype association to MVD severity obtained by the mixed linear model, additive, continuous variable analysis (EMMAX). The most significant regions of interest are noted on chromosome 15 and 2. Raw (P_raw)_ and Bonferroni corrected P values (P_adj_) are displayed for the top 2 SNPs. The red lines denote the level of genome wide significance (Bonferroni). B) The same manhattan plot is focused in on a region of chromosome 15 to demonstrate the flanking region of the most significant SNP. C) The same manhattan plot is focused in on a region of chromosome 2 to demonstrate the flanking region of the secondary peak of significance.

A total of 85,196 SNPs and 130 dogs remained for analysis after stringent quality control and exclusion of the X chromosome. Population stratification was evident based on visual inspection of the Q-Q plot and λ value = 1.07 (Fig 3A). The use of mixed linear model analysis was observed to return the Q-Q plot to the expected line and correct the mild population stratification previously observed (Fig 3B). Result of the EMMAX continuous variable, additive model, association analysis identified a SNP on chromosome 15 (SNP Chr15:58770326, *P*_raw_ = 5.84E-07, *P*_adjusted_ = 0.049) that was strongly associated with MVD severity score and genome-wide significant (Fig 4A). This SNP had a set of tightly clustered SNPs flanking its chromosomal location that approach but did not reach genome-wide significance (spanning Chr15:58506916–60140841 over 8 SNPs; P *P*_raw_ range of 1.68E-09–1.17E-05, Fig 4B, Table 2). This region contained multiple annotated genes as denoted in Table 3.

**Table 2:** The SNPs most significantly association with MVD in the continuous variable EMMAX analysis are detailed by location in CanFam 3.1 and test statistics.

Location	*P*_raw_-value	*P*_adjusted_-value
Chr 15: 58770326	5.84E-07	0.049
Chr 2: 37628875	9.36E-07	0.079
Chr 2: 37673511	9.36E-07	0.079
Chr 2: 37678327	9.36E-07	0.079
Chr 2: 37692930	9.36E-07	0.079
Chr 2: 37706121	9.36E-07	0.079
Chr 15: 58976835	1.49E-06	0.12
Chr 15: 59292603	1.68E-09	0.14
Chr 15: 59150571	1.77E-06	0.15
Chr 15: 58506916	2.82E-06	0.24
Chr 15: 59692840	3.15E-06	0.26
Chr 15: 58993573	5.01E-06	0.42
Chr 15: 58472645	7.22E-06	0.61
Chr 15: 60140841	1.17E-05	0.99

**Table 3:** Positional candidate genes are detailed by the abbreviation, product and location in CanFam3.1.

GENE	PRODUCT	CANFAM 3.1 LOCATION
FSTL5	follistatin-related protein 5 precursor	chr15:57985074–58670763
EEF1a1a	eukaryotic translation elongation factor 1 alpha 1	chr15:58853356–58854742
NAF1	H/ACA ribonucleoprotein complex non-core subunit NAF1 isoform a	chr15:59429553–59467422
NPY1R	neuropeptide Y receptor type 1	chr15:59580561–59583237
NPY5R	Neuropeptide Y receptor type 5	chr15:59606935–59608218
TMA16	Translation machinery associated protein	chr15:59720507–59750199
March1	E3 ubiquitin-protein ligase MARCH1	chr15:59754467–60036122
ARHGAP26	Rho GTPase-activating protein 26	chr2:37627422–38039759

Genotype association analysis identified a secondary tightly clustered region of 5 associated SNPs on chromosome 2 which were genome-wide suggestive but did not reach Bonferroni significance (Chr2:37628875, Chr2:37673511, Chr2:37678327, Chr2:37692930, Chr2:37706121, all *P*_raw_ = 9.36E-07 and *P*_adjusted_ = 0.079, Fig 4C, Table 2). Each of these SNPS was observed to reside within an intronic region of the ARHGAP26 gene (Table 3).

The case vs. control GWAS did not identify any significant SNPs with disease association.

## Discussion

Despite previous attempts, no convincing genome-wide association analysis results exist in dogs of any breed with MVD. While some reports identified loci associated with MVD, the phenotyping was questionable as identification of control samples was likely subjected to age-related errors and the results were not repeatable in other attempts [9, 11]. This is perhaps because the control subjects were too young to be considered absolute control dogs and their phenotyping was potentially not as robust as necessary [9, 11]. This historical information prompted us to develop a protocol by which every dog analyzed could be included in the analysis, thus improving power and reducing the necessity of a case versus control system.

Continuous variable GWAS is frequently used for quantitative traits. While development of MVD is not quantitative in the strictest sense of the definition, the echocardiographic parameters used to identify MVD are particularly amenable to establishing a scoring system, which facilitates this unique approach. Instead of phenotyping perfect control samples that were of very advanced age without evidence of disease, we elected to identify a genetic component of MVD linked to disease severity and age of onset. We believe that the high prevalence of this condition with advanced age, may argue that a causative disease variant is fixed in the breed and modifier loci may be responsible for observed expressivity differences.

We used findings that we considered supportive of diagnosing MVD and cumulative with disease severity to build a novel MVD severity score. Age-related penetrance of this condition necessitated the use of age in the development of the continuous variable. We reviewed data generated from the echocardiographic screenings and agreed that dogs 5 years of age or less with evidence of MVD were the most concerning for having a highly penetrant form of MVD. This age adjustment was also relevant as the median age in the study population. By accounting for this early age of onset, we developed the equation: (MVD severity score * 5) / patient’s age. A single continuous variable was generated by this equation for each sample and used to associate severity of MVD with genotype. Considering the novelty of this technique we sought the strictest criteria for determining genome-wide significance and utilized Bonferroni multiple testing corrections. Despite the robust correction, a region of significance was successfully identified.

The value of this approach is underscored by the loss of subjects that would meet inclusion criteria in the case vs. control GWAS, at least in part due to the confounding age-related penetrance. It is further supported by the lack of any significant regions of association in the case vs. control GWAS.

Clearly defining the severity of the echocardiographic parameters is crucial to the use of the proposed disease severity score. Parameters that required further definition include the severity of regurgitation and prolapse. We chose to include variable degrees of MR due to the knowledge that moderate to severe MR can exist in the absence of clinical signs and/or heart enlargement. This underscores the reasoning for assigning chamber enlargement a separate point on the disease severity scale [20]. The inclusion of mitral valve prolapse in the severity score is clinically relevant and multifactorial. In part it is based upon the information that severe mitral valve prolapse is associated with poor prognosis and thus more severe disease [21]. Additionally, the presence of mitral valve prolapse is present as an early indication of valvular disease in several breeds of dog [22–24].

Genes of interest were identified within the associated regions on chromosome 15 and within the suggestive region on chromosome 2. Further evaluation of these genes represents a possible next step in the molecular evaluation of MVD of Whippets. The positional candidate gene, FSTL5, has been previously identified as a prognostic marker in human patients with medulloblastoma [25]. Additionally, it was identified as a positional candidate gene in Irish Wolfhounds with dilated cardiomyopathy [26]. Although its functional pathway has not been studied in dogs and is not well elucidated in any species, it is a highly conserved gene from mammals to zebra fish to chickens. Gene ontology annotations report this gene to be involved in calcium ion binding (http://amigo.geneontology.org/amigo/term/GO:0005509). Interestingly, STRING_10_ (http://string-db.org/newstring_cgi/show_network_section.pl) evaluation of FSTL5 shows a single network connection in dogs to a protein, WFIKKN2 (WAP, follistatin/kazal, immunoglobulin, kunitz and netrin domain containing 2) that is reported to have metaloproteinase inhibition activity. This is of particular interest due to the reported matrix metalloproteinase involvement in the pathogenesis of human mitral valve prolapse and canine MVD [27].

ARHGAP26 (Rho GTPase-activating protein 26) is an activating protein with considerable interaction at the level of the extracellular matrix. It plays a role in the organization of actin-cytoskeleton and is expressed in many tissues, including the heart. Gene ontology annotations report this gene to be involved in phospholipid binding (http://amigo.geneontology.org/amigo/term/GO:0005543). Interestingly a gene paralog, ARHGAP21, was also identified as a candidate gene of interest in Irish Wolfhounds with dilated cardiomyopathy. The repeated overlap of these studies and the multiple hits identified in the evaluation of the cardiomyopathic Wolfhounds may suggest the success of our investigation in identifying genetic components of disease severity. Perhaps without intention the DCM loci of interest in this study were also markers of disease severity or early age of onset [26].

Ultimately this manuscript represents a novel approach to genome wide analysis for cardiac disease in dogs. The associated regions and positional candidate genes identified warrant further investigation through either candidate gene sequencing or next generation sequencing approaches. The use of GWAS to identify modifier genes or genetic loci associated with disease severity is a potential way to combat the phenotyping error that plagues studies of acquired disease of high frequency within the population.
